# The impact of COVID-19 on thyroid function and psychological state of Graves’ disease: a one-year prospective study

**DOI:** 10.3389/fendo.2025.1597083

**Published:** 2025-07-17

**Authors:** Lujia Xu, Jianbo Zhang, Riping Cong, Yujian Zhang, Xuenan Song, Wei Wang, Yingli Diao, Haijiao Liu, Kuanxiao Tang

**Affiliations:** ^1^ Department of General Practice, Qilu Hospital of Shandong University, Jinan, Shandong, China; ^2^ Department of Emergency and Chest Pain Center, Qilu Hospital of Shandong University, Jinan, Shandong, China; ^3^ Department of Internal Medicine, Jinan Hospital, Jinan, Shandong, China

**Keywords:** COVID-19, Graves’ disease, thyroid function, psychological state, medical intervention

## Abstract

**Background:**

Since the comprehensive lifting of coronavirus disease 2019 (COVID-19) pandemic control measures in mainland China in December 2022, the population has experienced widespread infection with COVID-19. COVID-19 affects multiple systems, including the endocrine system, particularly the thyroid. Graves’ disease, a common autoimmune disorder, may be complicated by COVID-19 infection. Therefore, investigating changes in thyroid function and psychological status in patients with Graves’ disease (GDC) and COVID-19 coinfection holds significant clinical importance.

**Methods:**

This study enrolled 110 hyperthyroid patients with COVID-19 coinfection, including 90 GDC patients meeting inclusion criteria. They were prospectively followed for one year at three time points: pre-COVID-19, 3 months, and 1 year post-infection. Patients were categorized by COVID-19 duration: G1 (≤5 days), G2 (6–8 days), and G3 (≥9 days). Follow-up included assessments of COVID-19 and GD symptoms, laboratory tests, psychological evaluations, treatment efficacy, COVID-19 management, and antithyroid medication adjustments. Statistical analyses (rank-sum tests, t-tests, multivariate logistic regression) explored COVID-19-GD associations and changes in thyroid function and psychological status in GDC patients.

**Results:**

Multivariate logistic regression analysis, after covariate adjustment, identified the number of COVID-19 symptoms as an independent risk factor for hyperthyroidism in GDC patients, and COVID-19 duration as an independent risk factor for poor psychological status. At 3 months post-infection, the G3 group showed an increased FT3/FT4 ratio and decreased FT4 levels. Significant intergroup differences were observed in FT4 and TSH changes from pre-infection levels, with the G3 group having the highest anxiety and depression scores. Antithyroid medication and psychological interventions were adjusted based on thyroid function and psychological scores. At 1 year post-infection, TSH levels in the G1 and G3 groups increased compared to 3 months, while psychological scores decreased. The G3 group had significantly higher TSH levels than pre-infection, with significant intergroup differences in FT3 and FT4 levels.

**Conclusion:**

Post-COVID-19 infection, GDC patients may experience hyperthyroidism and psychological distress, which improve with tailored ATD adjustments and psychological interventions. The FT3/FT4 ratio guides (antithyroid drugs) ATDs optimization, while psychological intervention effectively mitigates anxiety and depression in GDC patients.

## Introduction

Coronavirus disease 2019 (COVID-19) was declared a pandemic by the World Health Organization on March 11th, 2020, which seriously threatened human health (1). Current literature indicates that COVID-19 can induce thyroid dysfunction (hyperthyroidism or hypothyroidism) in patients without pre-existing thyroid disease. COVID-19 can lead to hyperthyroidism, hypothyroidism, Hashimoto thyroiditis, etc. This study mainly studies the changes of thyroid function and psychological status in patients with Graves’ disease (GD) after infection with COVID-19. However, there is a lack of studies investigating thyroid function changes in GD patients following COVID-19 infection. As an autoimmune disease, GD, like other autoimmune diseases, highly inflammatory diseases related to severe SARS-CoV-2 infection may trigger the immune cascade reaction of GD (2–5). Fully lifting COVID-19 pandemic control measures in mainland China in 12/2022 had made the incidence of COVID-19 increased markedly, so that GD with COVID-19 (GDC) patients could not being able to receive timely follow-up care (6). Although the respiratory system is the primary target organ of COVID-19 infection (7), extrapulmonary organs may also be affected by COVID-19 infection (8). Studies have shown that COVID-19 might trigger GD recurrence (9). A national observational study shows that the mortality rate of COVID-19 patients with GD is significantly increased (OR 1.54, 95% CI, 1.02–2.33, ***P*** = 0.042) (9). The research has shown that the severity of COVID-19 infection in patients is related to their thyroid function (9). After the onset of COVID-19 symptoms, there have been reported cases of GD recurrence or deterioration (10). In addition, the COVID-19 Mental Disorders Collaborators concluded that the pandemic led to an increase in cases of major depressive disorders and increase in cases of anxiety disorders globally (11). Due to the patients’ unfamiliarity with the new pandemic infectious disease and psychological fear of infection, as well as sudden isolation from family and society due to epidemic prevention measures during the outbreak, these unexpected circumstances have imposed significant mental and physical burdens on the patients (12). However, the impact of the duration and number of COVID-19 symptoms on thyroid hormone levels and mental state was not explained. Therefore, it is necessary to clarify the changes in thyroid hormone levels and psychological state of GDC patients. And the effect of suitable psychological intervention and the adjustment of antithyroid drugs (ATDs) on GDC patients needs to be investigated.

## Materials and methods

### Participants

110 hyperthyroidism with COVID-19 (HTC) patients were enrolled, and 90 GDC patients were finally followed up for one year at Qilu Hospital of Shandong University. Inclusion criteria: (1) Age ≥ 18 years; (2) Patients in the maintenance phase of Graves’ disease (GD) treatment, as defined by the Chinese Guidelines for the Diagnosis and Treatment of Hyperthyroidism and Other Causes of Thyrotoxicosis (13). (3) Meeting the diagnostic criteria for mild COVID-19 as per the Diagnosis and Treatment Protocol for Novel Coronavirus Pneumonia (Trial Version 10) issued by the National Health Commission (14). Exclusion criteria: (1) Incomplete medical records or loss to follow-up; (2) Pregnant or lactating women; (3) Comorbid severe underlying diseases or immune-related disorders; (4) Recent or ongoing special treatments that may affect thyroid function or immune status. All patients were treated with methimazole (MMI), and no antiviral medications were administered.

**Figure 1:**
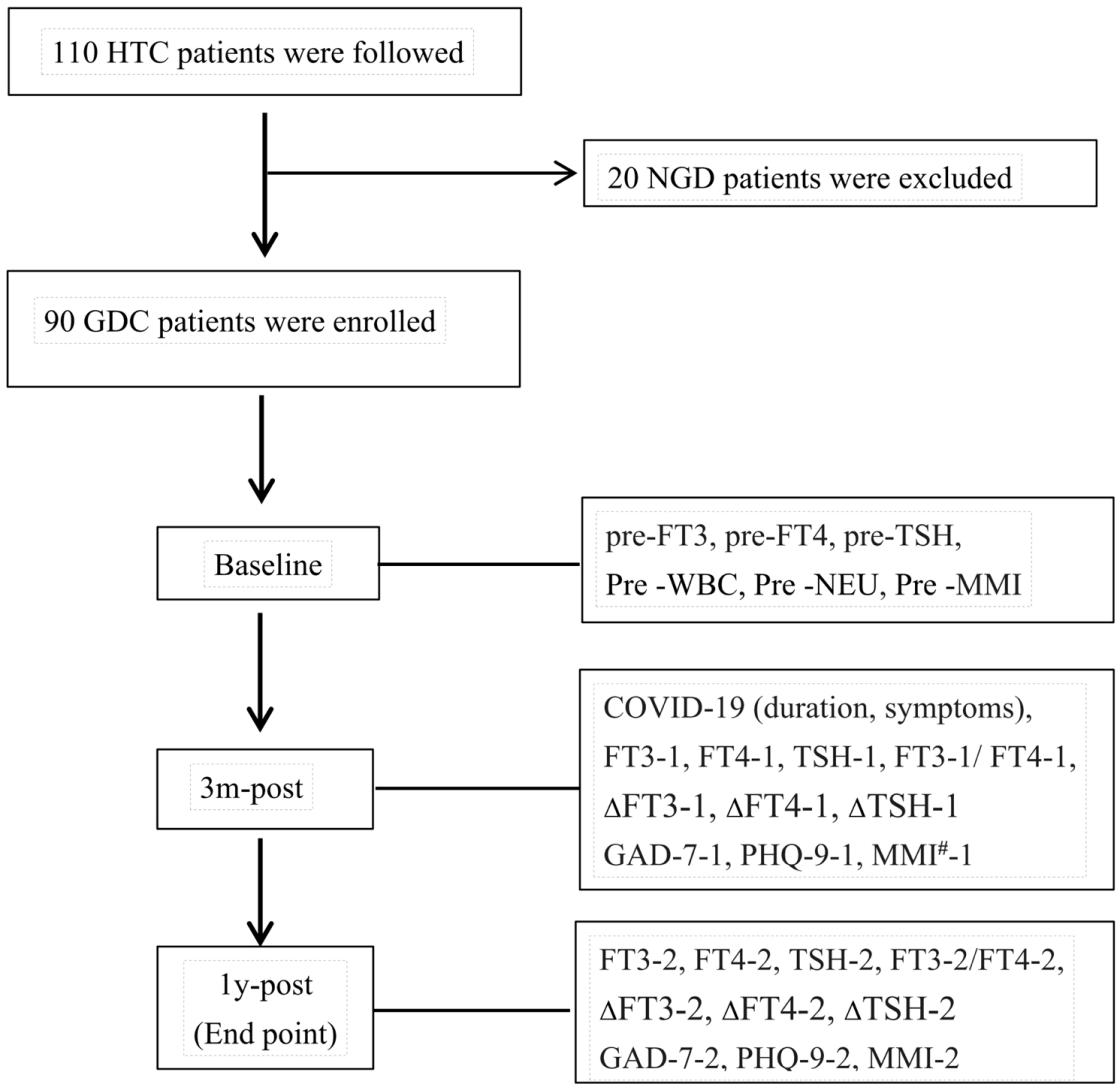
HTC: patients with hyperthyroidism complicated by COVID-19. NGD: non-GD patients with comorbid hyperthyroidism. 3m-post, 1-post, at 3 months, 1 year post-COVID-19.

This study is a prospective investigation with follow-up time points at pre-COVID-19, 3 months post-COVID-19 infection, and 1 year post-COVID-19 infection, as illustrated in the **Figure 1**.

Studies have shown that the median duration of IgM and IgA antibody detection was 5 days (IQR, 3-6) (15), and the mean duration of symptoms was approximately 8.89 days, with a median duration of 8.0 days (16). Therefore, patients were divided into three groups based on the duration of COVID-19: Group 1 (G1) ≤ 5 days, Group 2 (G2) 6–8 days, and Group 3 (G3) ≥ 9 days.

The relevant clinical data collected during follow-up included:

1. COVID-19-related data: Duration of illness (days), symptoms (e.g., fever, dry cough), physical signs, and treatment details.
2. Graves’ disease-related data: Duration of GD treatment, symptoms (e.g., sweating, palpitations, irritability, increased appetite, weight loss), physical signs (e.g., goiter, exophthalmos, fine hand tremors), thyroid function (e.g., FT3, FT4, TSH), and antithyroid drugs (ATDs) dosage.
3. Psychological assessment and intervention: Scores from psychological assessment scales for GDC patients and methods of psychological intervention.

The ethics committee of Qilu Hospital of Shandong University permitted this study following the guidelines and regulations (KYLL-202307-049). Informed consent was obtained before the follow-up.

### GAD-7 and PHQ-9

In this study, anxiety and depression levels among GDC patients were assessed using both the Generalized Anxiety Disorder-7 (GAD-7) and Patient Health Questionnaire-9 (PHQ-9) scales. A dedicated member of the research team conducted face-to-face psychological evaluations with patients, administering the questionnaires and recording responses. The complete questionnaires and scoring sheets are provided in **Supplementary Tables S1**, **S2**.

### Measurement of thyroid function

Serum levels of FT3, FT4, and TSH were quantitatively measured using electrochemiluminescence immunoassay (ECLIA) at the clinical laboratory of Qilu Hospital, Shandong University. The normal reference ranges for serum thyroid function tests are as follows: FT3: 3.1–6.8 pmol/L, FT4: 12–22 pmol/L, thyroid-stimulating hormone (TSH): 0.27–4.2μIU/mL. All participants were clinically stable Graves’ disease patients in the maintenance phase, demonstrating euthyroid status and stable mental health parameters without outliers.

### Statistical analysis

Statistical analyses were performed using IBM SPSS Statistics. Normality of continuous variables was evaluated using the Shapiro-Wilk test. Data following a normal distribution were expressed as mean ± standard deviation (SD) and analyzed using paired t-tests for within-group comparisons or independent t-tests for between-group comparisons. Non-normally distributed data were reported as median (interquartile range, IQR) and analyzed with the Wilcoxon signed-rank test (within-group) or Kruskal-Wallis test (between-group). Categorical variables were presented as frequencies (percentages) and compared using chi-square or Fisher’s exact tests. Univariate logistic regression was employed to identify potential risk factors affecting thyroid function and psychological status in GDC patients. Multivariate logistic regression was subsequently performed to adjust for confounders and determine independent risk factors, with outcomes reported as odds ratios (OR) and 95% confidence intervals (CI). All analyses were two-tailed, with statistical significance set at ***P*** < 0.05.

## Results

### Baseline characteristics of GDC patients

This study followed 110 hyperthyroid patients with COVID-19. After applying inclusion and exclusion criteria, 90 Graves’ disease (GD) patients in the maintenance treatment phase were enrolled. All patients denied a history of chronic conditions such as hypertension, coronary artery disease, diabetes, rheumatic immune diseases, or psychiatric disorders. None used antiviral medications during COVID-19 infection, and all abstained from smoking, alcohol, adhered to a low-iodine diet, and attended regular follow-ups. There were no significant differences among the three GD patient groups in age, sex ratio, baseline thyroid function levels, liver function, blood routine parameters, or antithyroid drug (ATD) doses (***P*** >0.05) (**Table 1**).

**Table 1:** Characteristics of GDC patients in baseline (n = 90).

	G1 (n=34)	G2 (n=34)	G3 (n=22)	*P*
Age	39.27 ± 13.89	38.27 ± 13.84	39.91 ± 13.40	0.90
Gender(M/F)	9/25	7/27	5/17	0.90
Course of GD(years)	3.00 (2.00, 6.00)	3.75 (2.00, 5.00)	3.5 (1.00, 5.00)	0.10
Pre-FT3(pmol/L)	4.85 (4.34, 5.75)	4.70 (4.24, 5.01)	4.57 (4.12, 6.42)	0.20
Pre-FT4(pmol/L)	14.55 (13.17, 17.75)	13.10 (11.10, 16.65)	17.63 (14.48, 21.49)	0.10
Pre-FT3/FT4	0.33 (0.30, 0.38)	0.35 (0.29, 0.42)	0.28 (0.24, 0.35)	0.30
Pre-TSH(μIU/ml)	2.27 (0.58, 4.03)	2.23 (0.58, 4.03)	1.29 (0.29, 3.07)	1.00
Pre-MMI(mg/day)	5.00 (0.94, 10.00)	5.00 (0.94, 10.00)	2.50 (0.25, 11.25)	0.90
Pre-WBC(10^9^/L)	6.12 ± 1.37	6.00 ± 1.54	6.30 ± 1.72	0.77
Pre-NEU%	54.50 (48.60, 59.08)	54.85(48.25, 57.40)	54.25 (49.76, 58.75)	0.94
Pre-NEU(10^9^/L)	3.33 ± 1.19	3.29 ± 1.14	3.41 ± 1.14	0.97
Pre-LYM%	35.25 (31.18, 40.30)	38.05 (32.28, 42.80)	38.70 (30.40, 42.61)	0.35
Pre-LYM(10^9^/L)	2.18 ± 0.52	2.16 ± 0.61	2.39 ± 0.75	0.54
Pre-ALT(U/L)	14.00 (11.00, 17.00)	12.00 (10.00, 16.50)	15.00 (13.00, 22.25)	0.19
Pre-AST(U/L)	15.00 (13.75, 18.00)	16.50 (14.75, 19.00)	17.00 (14.75, 21.75)	**0.02**

Data following a normal distribution were expressed as mean ± SD, non-normally distributed data were reported as media (IQR)

GD: Graves’ disease, Pre-FT3, Pre-FT4, Pre-TSH: Serum levels of free Triiodothyronine, free thyroxine, thyroid-stimulating hormone before COVID-19, Pre-MMI daily dosage of methimazole before COVID-19. Pre-WBC, Pre-NEU%, Pre-NEU, Pre-LYM%, Pre-LYM: white blood cell count, neutrophil ratio prior, neutrophil count, lymphocyte ratio and lymphocyte count prior to COVID-19. Pre-ALT, Pre-AST: Serum levels of alanine aminotransferase and aspartate aminotransferase befpre COVID-19.Bold font indicates ***P*** <0.05, which is statistically significant.

### Comparison of the number of COVID-19 symptoms among the 3 groups

**Figure 2:**
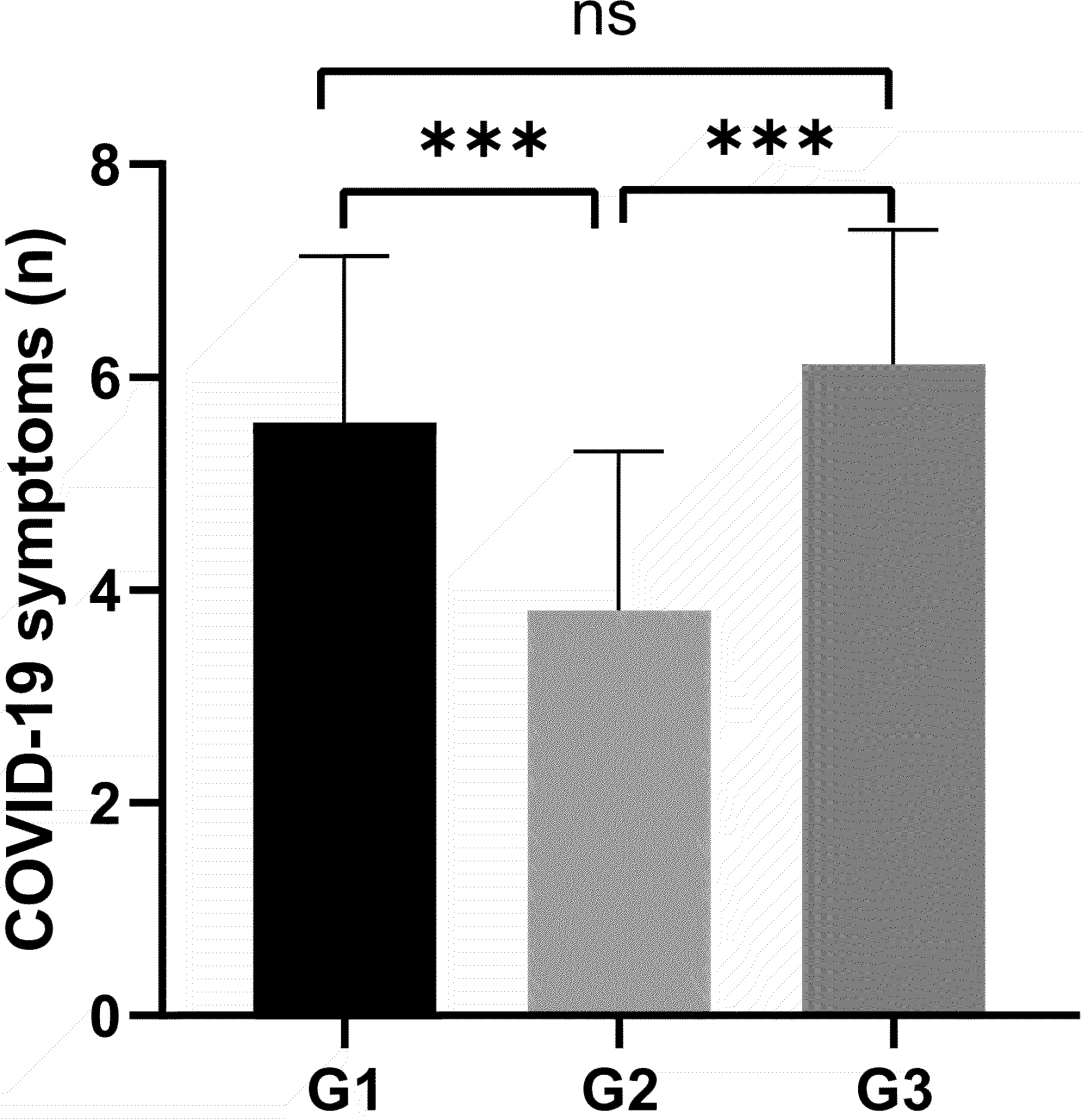
All variables plotted with bar indicating mean with SD. All patients were classified by the duration of COVID-19 G1: ≤5 days, G2: 6-8 days, G3: ≥9days. The height of the column represents the average, and the distance from the top of the column to the upper line of the column represents the standard deviation (SD). Variance analysis used for comparison of above variable. ***P < 0.001. ns: no significance.

**Figure 2** demonstrates a statistically significant difference in the number of COVID-19 symptoms among different groups of GDC patients. The G1 group exhibited fewer symptoms (5.6 ± 1.5), the G2 group had the fewest (4 ± 1.5), and the G3 group showed the highest number of symptoms (6 ± 1.5), with statistical significance (***P*** <0.001). These findings provide critical insights for investigating the impact of COVID-19 on thyroid function in GDC patients.

### Changes of thyroid function and psychological state in GDC patients after diagnosing COVID-19 for three months

**Figure 3:**
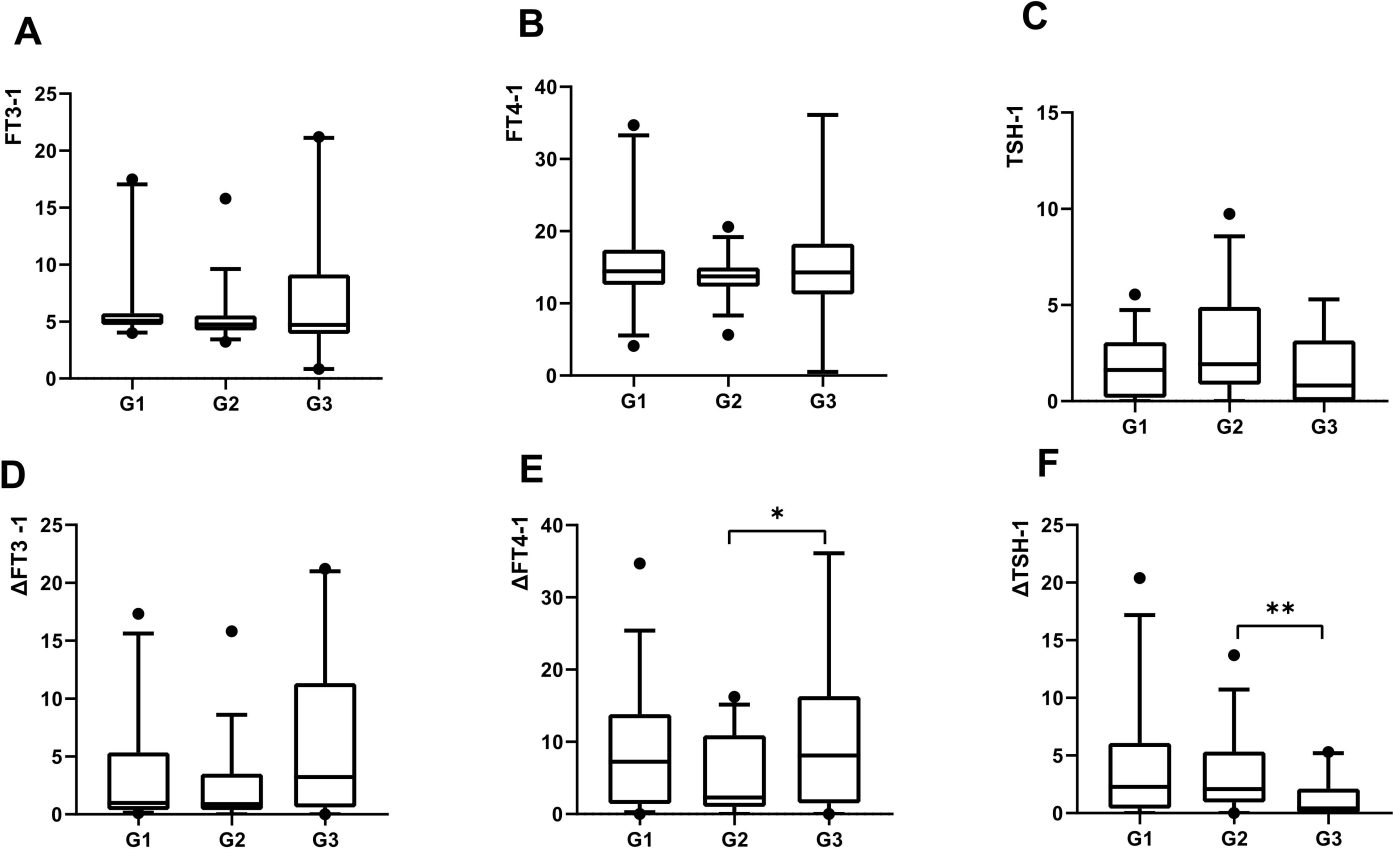
All variables plotted with box indicating 25th and 75th percentiles, whiskers indicating fifth and 95th percentiles, and line in box indicating median. All patients were classified by the duration of COVID-19: G1, 5 days; G2, 6-8 days, G3, ≥9days.The level of serum FT3 at 3months post-COVID-19 were demonstrated by A. The level of serum FT4 at 3months post-COVID-19 were demonstrated by B. The level of serum TSH at 3months post-COVID-19 were demonstrated by C. The change of serum FT3 at 3months post-COVID-19 were demonstrated by D. The change of serum FT4 at 3months post-COVID-19 were demonstrated by E. The change of serum TSH at 3months post-COVID-19 were demonstrated by F. Kruskal-Wallis used for comparison of above variables. *P < 0.05, **P < 0.01.

Three months after COVID-19 infection, the ratio of serum free triiodothyronine to serum free thyroxine (FT3-1/FT4-1) significantly increased in the G3 group (***P*** = 0.015). The changes in FT4 (pmol/L) and thyroid-stimulating hormone (TSH) levels (ΔFT4-1, ΔTSH-1, defined as the difference between pre-COVID-19 and 3 months post-infection) were significantly different among the three groups [G1: 7.94 (1.60, 14.93); G2: 2.30 (1.11, 11.10); G3: 8.62 (2.03, 21.83), ***P*** =0.03; G1: 2.28 (0.40, 6.09); G2: 2.09 (0.94, 5.34); G3: 0.41 (0.005, 2.13), ***P*** = 0.01] (**Figure 3**).

**Figure 4:**
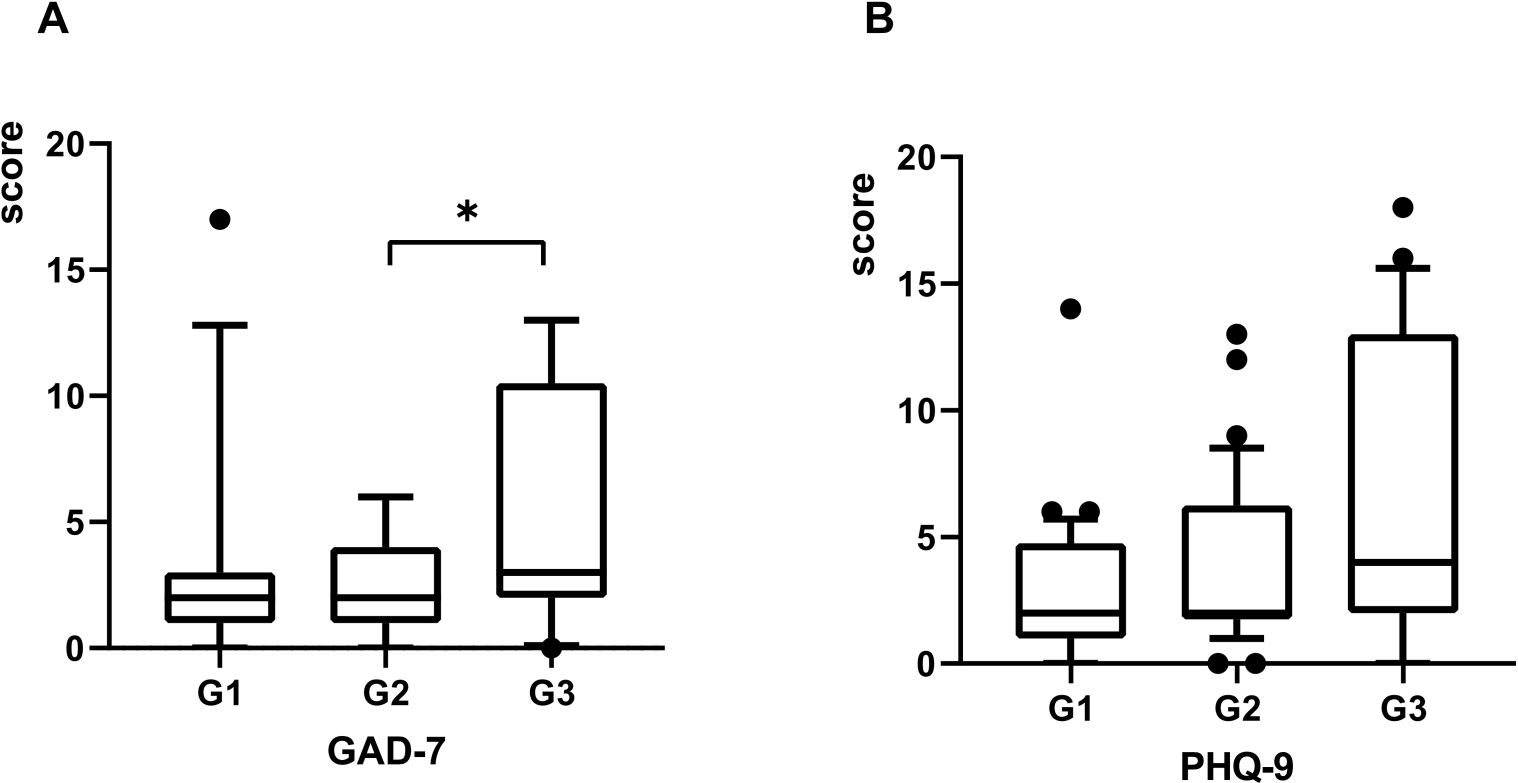
The scores of GAD-7 and PHQ-9 plotted with box indicating 25th and 75th percentiles, whiskers indicating fifth and 95thpercentiles, and line in box indicating median. The scores of GAD-7 and PHQ-9 at 3 months post-COVID-19 were demonstrated by **(A, B)**. Kruskal-Wallis rank-sum tests used for comparison, *P < 0.05.

Psychiatric comorbidities are prevalent among GD patients (17, 18). All three patient groups exhibited symptoms of anxiety and depression following COVID-19 infection, with the G3 group demonstrating the highest scores on both the GAD-7 and PHQ-9 scales (**Table 2**). Significant differences in GAD-7 scores were observed across the groups, with *post-hoc* analysis revealing a statistically significant difference specifically between G2 and G3 (***P*** = 0.002) (**Figure 4**). In contrast, no significant differences in PHQ-9 scores were identified among the three groups (**Figure 4**).

**Table 2:** Changes of GAD-7 and PHQ-9.

Time points	Scale	Group	Median (P25, P75)
3m-post	GAD-7	G1	2 (1, 3.25)
		G2	2 (1, 4)
		G3	3 (2, 10.5)
	PHQ-9	G1	2 (1, 3.5)*
		G2	2 (1.75, 6.25)
		G3	4 (2, 13)
1year-post	GAD-7	G1	0 (0, 4)^&^
		G2	0 (0, 1)^&^
		G3	0 (0, 1)^&^
	PHQ-9	G1	0 (0, 4)
		G2	0 (0, 2) ^&^
		G3	0 (0, 1.5) ^&^

3m-post,1-post, at 3 months, 1 year post-COVID-19, GAD-7, the 7-item Generalized Anxiety Disorder, PHQ-9, the 9-item Patient Health Questionnaire depression module.

^*^ *P* < 0.05, comparison among three groups at 3 months post-COVID-19.

^&^ *P* < 0.05, comparison between 3 months and 1 year post-COVID-19 infection in every group.

### Changes of thyroid function and psychological state in GDC patients after recovering from COVID-19 for one year

One year after COVID-19 infection, serum TSH levels (μIU/mL) in GDC patients from the G1 and G3 groups were significantly higher compared to levels at 3 months post-infection [G1:.63 (0.18, 3.06) *vs* 2.60 (1.47, 3.70), ***P*** <0.05;G3: 0.81 (0.01, 3.15) *vs* 4.64 (1.92, 7.84), ***P*** <0.05]. Compared to pre-COVID-19 levels, no significant changes in serum TSH were observed in the G1 and G2 groups (***P*** > 0.05), while a significant increase was noted in the G3 group (***P*** < 0.05). In the G3 group, the changes in serum FT3 and FT4 levels (pmol/L) (ΔFT3–2 and ΔFT4-2, representing the difference between 3 months and 1 year post-COVID-19 infection) showed statistically significant differences compared to 3 months post-infection (P < 0.05) (**Table 3**).

**Table 3:** Changes of the level of thyroid function in the two follow-up visits.

Time points	Group	FT3	FT4	FT3/FT4	TSH	ΔFT3	ΔFT4	ΔTSH
Base	G1	4.85 (4.34, 5.75)	14.55 (13.17, 17.75)	0.33 (0.30, 0.38)	2.27 (0.58, 4.03)	‐	‐	‐
	G2	4.70 (4.24, 5.01)	13.10 (11.10, 16.65)	0.36 (0.30, 0.43)	2.23 (1.37, 6.15)	‐	‐	‐
	G3	4.57 (4.12, 6.42)	17.63 (14.48, 20.75)	0.28 (0.24, 0.35)	1.29 (0.29, 3.07)	‐	‐	‐
3m-post	G1	5.10 (4.70, 5.75)	14.70 (12.73, 18.25)	0.35 (0.30, 0.45)	1.63 (0.18, 3.06)	0.98 (0.38, 5.35)	7.94 (1.60, 14.93)	2.28 (0.40, 3.06)
	G2	4.75 (4.23, 5.23)	13.80 (12.34, 15.25)	0.40 (0.23, 0.59)	1.63 (0.18, 3.06)	0.89 (0.38, 3.51)	2.30 (1.11, 11.10)	1.92 (0.86, 4.90)
	G3	4.70(4.00, 12.90)	15.70 (12.50, 27.30)*	0.41 (0.33, 0.56)*	0.81 (0.01, 3.15)	3.78 (0.68, 14.19)	8.62 (2.03, 21.83)^#^	0.41 (0.01, 2.13)^#^
1y-post	G1	5.01 (4.38, 5.50)	15.00 (14.10, 17.10)	0.34 (0.30, 0.38)	2.60 (1.47, 3.70)*	2.53 (0.43, 5.35)	5.56 (2.43, 16.38)	2.25 (1.05, 3.36)
	G2	5.22 (4.78, 5.80)	15.10 (13.79, 17.30)	0.33 (0.29, 0.43)	2.20 (1.27, 3.37)	0.92 (0.44, 1.83)	15.10 (13.79, 17.30)	1.83 (0.98, 3.41)^&^
	G3	5.12 (4.76, 6.27)	15.60 (13.40, 16.73)	0.41 (0.33, 0.56)	4.64 (1.92, 7.84)*	2.97 (0.94, 9.72)	8.42 (2.18, 21.28)	3.50 (0.16, 6.19)^&^

3m-post,1-post, at 3 months, 1 year post-COVID-19, FT3, free triiodothyronine; FT4, free thyroxine; TSH, thyroid stimulating hormone; ΔFT3, the change of FT3; ΔFT4, the change of FT4; ΔTSH, the change of TSH.

^*^ *P <*0.05, in the G3 group, there were statistically significant changes in FT4 levels and the FT3/FT4 ratio between pre-COVID-19 infection and 3 months post-COVID-19 infection,between 3 months post-COVID-19 infection and1 year post-COVID-19 infection.

^&^ *P* < 0.05, a statistically significant difference in serum TSH levels between 3 months and 1 year post-COVID-19.

^#^ *P* < 0.05,at 3 months post-COVID-19 infection, there were statistically significant differences in ΔFT4 and ΔTSH among the different groups).

**Figure 5:**
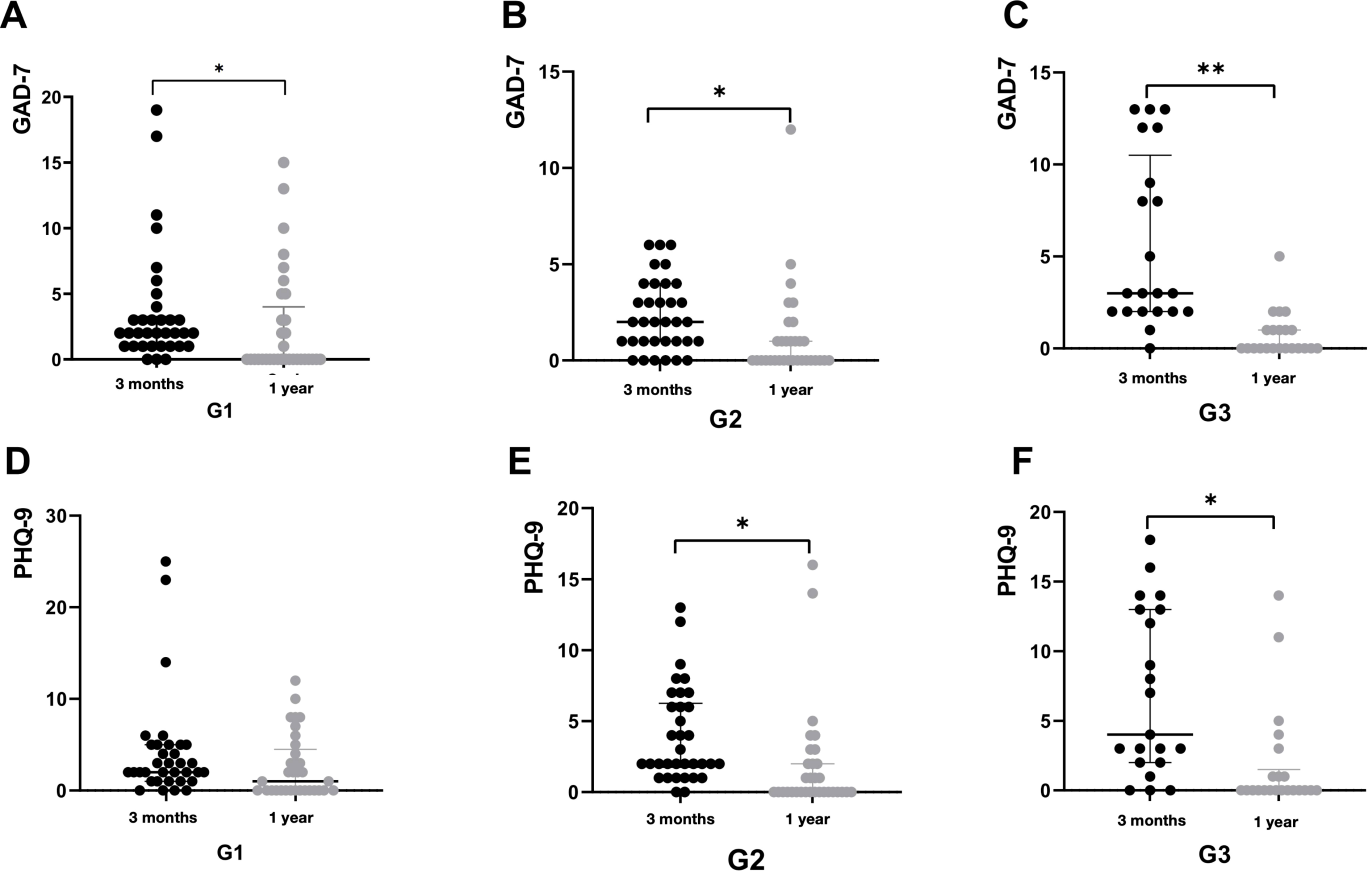
The score of psychological scale at 1 year post-COVID-19, comparing to that at 3 months post-COVID-19 were demonstrated by figure 5. All patients were classified by the duration of COVID-19: G1, 5 days; G2, 6-8 days, G3, ≥9days. The score of GAD-7 at 3months and 1year post-COVID-19 in G1 were demonstrated by A. The score of GAD-7 at 3months and 1year post-COVID-19 in G2 were demonstrated by B. The score of GAD-7 at 3months and 1year post-COVID-19 in G3 were demonstrated by C. The score of PHQ-9 at 3months and 1year post-COVID-19 in G1 were demonstrated by D. The score of PHQ-9 at 3months and 1year post-COVID-19 in G2 were demonstrated by E. The score of PHQ-9 at 3months and 1year post-COVID-19 in G3 were demonstrated by F. *P < 0.05.

After Solution-focused Brief Therapy (SFBT), the patients’ psychological state significantly improved. There were no significant differences in the effects among the three groups (**Table 2**). However, compared with the psychological scale scores at the first follow-up, the scores of the three groups of patients decreased significantly, especially the GAD-7 scores (**Table 2**, **Figure 5**).

### Correlation analysis between COVID-19 and thyroid function as well as psychological scores in GDC patients

**Figure 6:**
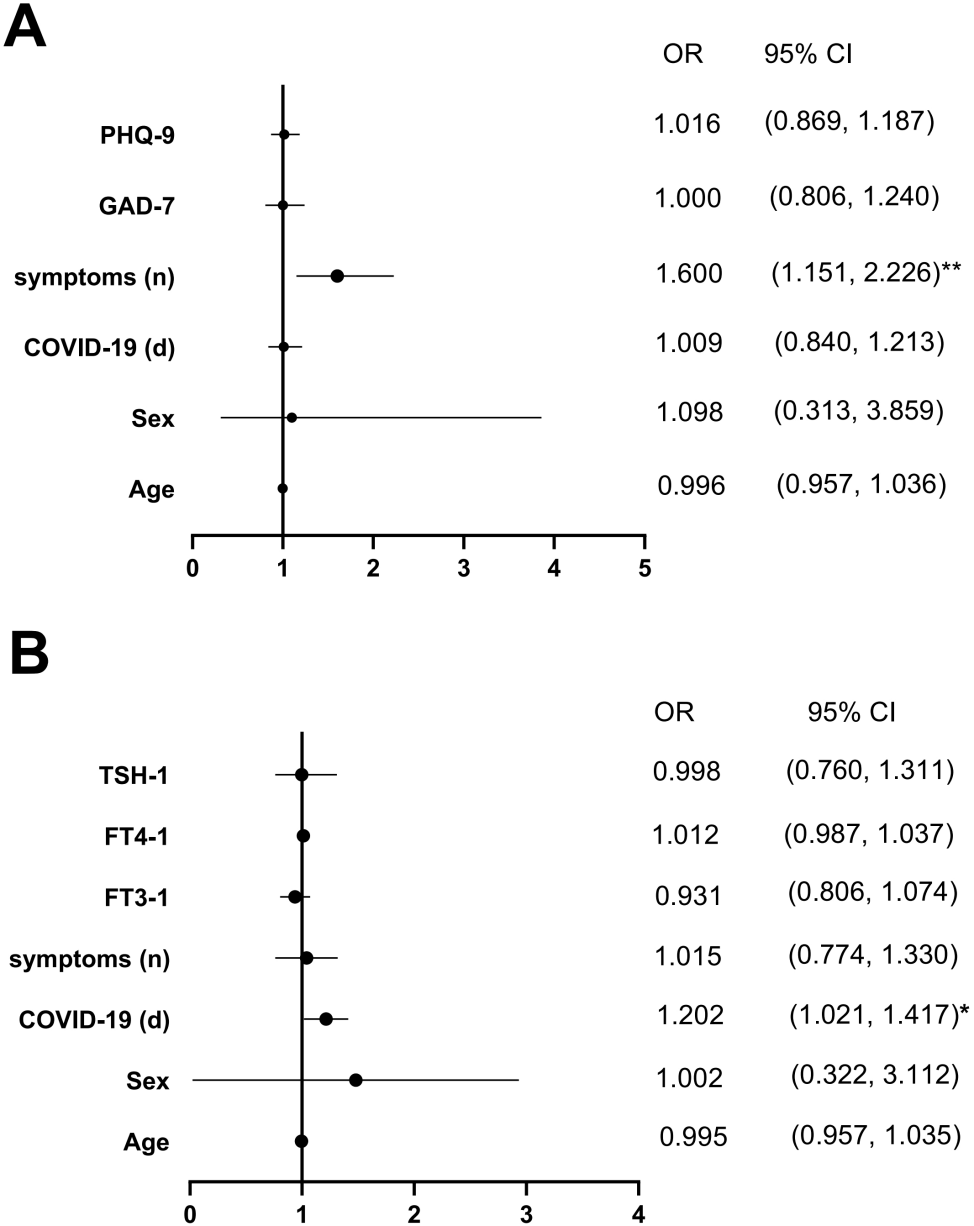
Multivariate-adjusted logistic regression model of the association between FT3/FT4 and COVID-19, psychological state and Psychological state after the first follow-up were demonstrated by **(A, B)**. Abbreviations: FT3-1, FT4-1, TSH-1, the levels of FT3, FT4 and TSH; *P < 0.05, **P < 0.01.

Univariate logistic regression analysis indicated that age, sex, duration of COVID-19, number of COVID-19 symptoms, PHQ-9 score, and GAD-7 score were significantly associated with changes in the FT3/FT4 ratio (***P*** < 0.05). Using the change in the FT3/FT4 ratio as the dependent variable (with differences between FT3-1/FT4–1 and pre-FT3/pre-FT4 ratios < 0 coded as 0 and > 0 as 1), multivariate logistic regression revealed that the number of COVID-19 symptoms was an independent predictor of an increased FT3/FT4 ratio (***P*** < 0.01) (**Figure 6**).

Similarly, age, sex, serum TSH-1, FT3-1, FT4–1 levels, number of COVID-19 symptoms, and duration of COVID-19 (days) were associated with anxiety severity in GDC patients (***P*** < 0.05). Using GAD-7 scores (< 5 coded as 0, ≥ 5 as 1) as the dependent variable and the aforementioned factors as independent variables, multivariate logistic regression identified the duration of COVID-19 (days) as an independent predictor of anxiety severity (***P*** < 0.05) (**Figure 6**).

### Adjustment of ATD dosage in GDC patients

At 3 months post-COVID-19 infection, the methimazole (MMI) dosage in GDC patients from the G1, G2, and G3 groups was significantly increased compared to pre-infection levels (***P*** < 0.001, ***P*** < 0.01, and ***P*** < 0.001, respectively). In the G1 group, the MMI dosage at 1 year post-infection showed no significant change compared to 3 months post-infection (***P*** > 0.05) but remained higher than the pre-infection dosage (***P*** < 0.05), suggesting that some patients still required higher MMI doses to maintain GD stability 1 year after COVID-19 infection. In contrast, the MMI dosage in the G2 and G3 groups decreased at 1 year post-infection compared to 3 months post-infection (***P*** < 0.05 and ***P*** < 0.001, respectively) (**Table 4**).

**Table 4:** Comparison of MMI dosages in GDC patients.

Group	pre-MMI (mg/d)	MMI-1(mg/d)	MMI-2(mg/d)
G1	3.75 (0.00, 10.00)	5.00 (2.50, 10.00) ***	5.00 (2.19, 10.00) ^#^
G2	5.00 (1.25, 10.56)	5.00 (2.50, 15.00) **	5.00 (1.25, 10.00) ^&^
G3	5.00 (1.88, 10.63)	10.00 (5.00, 26.25) ***	5.00 (2.50, 13.13) ^&&&^

***P < 0.001; **P < 0.01: The difference in MMI dosage between pre-COVID-19 infection and 3 months post-COVID-19 infection in GDC patients was statistically significant. ^#^P < 0.05: The difference in MMI dosage between pre-COVID-19 infection and 1 year post-COVID-19 infection in GDC patients was statistically significant. ^&^P < 0.05, ^&&&^P < 0.001: The difference in MMI dosage between 3 months post-COVID-19 infection and 1 year post-COVID-19 infection in GDC patients was statistically significant.

pre-MMI, MMI-1, and MMI-2: MMI dosages before COVID-19 infection, at 3 months post-COVID-19 infection, and at 1 year post-COVID-19 infection, respectively.

## Discussion

This study investigated the impact of COVID-19 on thyroid function and the psychological state of GD patients. After diagnosing COVID-19 for three months, the ratio of FT3-1/FT4–1 increased, the serum level of FT4 decreased, and ΔFT4–1 and ΔTSH-1 changed significantly. Moreover, GDC patients often exhibit a noticeable decline in their psychological well-being, particularly experiencing heightened anxiety. After the adjustment of MMI dosage and SFBTt in GDC patients, the above impacts improved after one year of COVID-19 infection.

Previous studies have indicated that COVID-19 may lead to thyroid dysfunction in patients (17–19). Studies have shown that the severity of COVID-19 is related to thyroid function (14). This study found that the above conclusions still apply to patients with GD. Therefore, early detection of thyroid function in patients with GDC is particularly important and needs close monitoring.

Studies have shown that ratio of FT3/FT4 reacts 5’-deiodinase activity, which can be used to evaluate peripheral thyroid sensitivity (20–22). There was a significant difference in FT3-1/FT4-1, especially in G3. the ratio of FT3-1/FT4-1, FT4, ΔFT4–1 and ΔTSH-1 changed the most when COVID-19 had the longest duration and most symptoms after diagnosing COVID-19 for three months. Studies have shown that thyroid function deteriorates briefly in COVID-19, and routine measurement of thyroid function in COVID-19 may not be necessary (23). However, routine measurement of thyroid function and the adjustment of MMI in GD patients are necessary. The change in thyroid hormone levels after COVID-19 leads to an increase in MMI dosage, particularly in patients with a longer duration and more symptoms of COVID-19. As thyroid function improved, the dosage of MMI decreased after one year. Hence, it is essential to monitor the thyroid function of GDC patients and then make appropriate adjustments to MMI dosage.

Studies had shown that GD patients were prone to anxiety and depression (24, 25). Research indicates when hyperthyroidism occurs, excessive secretion of thyroid hormone affects brain regions such as amygdala, hippocampus, frontal cortex, and then affects nervous system function, making people prone to tension, anxiety, irritability and other emotions. Research also showed that patients with COVID-19 were more prone to experiencing anxiety and depression (26). SFBT is highly compatible with patients suffering from hyperthyroidism after COVID-19, and it can effectively address the psychological issues of these patients (27). After fully lifting COVID-19 pandemic control measures, if COVID-19 persists and presents more symptoms, both GAD-7 and PHQ-9 showed the highest scores. This indicated that COVID-19 had a long duration and more symptoms, which might be more likely to cause anxiety and depression after diagnosing COVID-19 for three months. After SFBT, the patients experienced a significant reduction in anxiety and depression. Hence, GDC patients also require the same psychological intervention measures.

Multivariate regression analysis revealed that the duration of COVID-19 and the number of COVID-19 symptoms were the sole influencing factors on the thyroid function and psychological state of GDC patients respectively. The longer the duration of COVID-19, the more symptoms it has, and the GDC patients are more likely to experience thyroid function deterioration and poor psychological state. However, the thyroid function and psychological state of GDC patients recovered after one year with adjusting the treatment plan. Therefore, it is necessary to adjust the dose of MMI based on the duration of COVID-19 and the number of COVID-19 symptoms in GDC patients, and to implement suitable psychological interventions (28).

However, under the background of COVID-19 pandemic, there are some limitations in this study: (1) Fully lifting COVID-19 pandemic control measures had resulted in a relatively small sample size and a lack of control group. (2) The cases included in this study were outpatients, and the county official indicators such as inflammatory factors were not detected, so the impact of inflammatory response on psychiatric comorbidities could not be accurately judged. (3) The patient’s condition is affected by the sealing of epidemic holes and traffic factors, but due to the special period of the epidemic, these factors cannot be specifically investigate. However, we believe our data are sufficient to suggest that the impact of COVID-19 on thyroid function and the psychological state had basically recovered through appropriate adjustment treatment.

## Conclusion

GDC patients may develop hyperthyroidism and adverse psychological states post-COVID-19. Adjusting ATDs and psychological interventions improve thyroid function and mental health. COVID-19 can cause anxiety and depression in GDC patients, with symptom count being an independent risk factor for thyroid function changes. The FT3/FT4 ratio guides ATD dosage adjustment, and SFBT effectively alleviates anxiety and depression in GDC patients.

## Data Availability

The raw data supporting the conclusions of this article will be made available by the authors, without undue reservation.
