# Supplementary figures and images for: The impact of COVID-19 on thyroid function and psychological state of Graves’ disease: a one-year prospective study
Source: Front Endocrinol (Lausanne). 2025 Jul 17;16:1597083. doi: 10.3389/fendo.2025.1597083 (PMC12310469; doi:10.3389/fendo.2025.1597083)

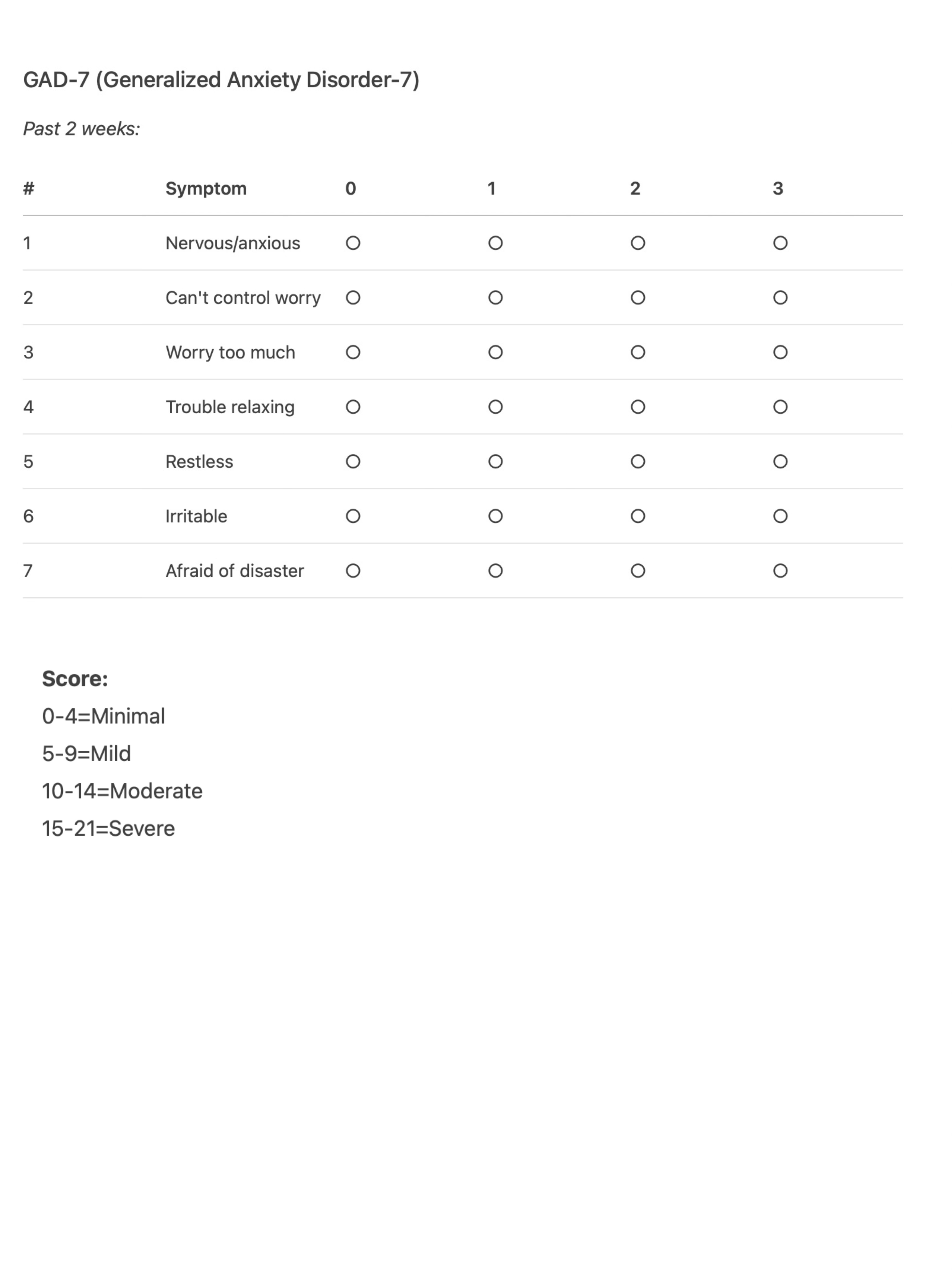

Supplement: Supplementary file 1 [file SupplementaryFile1.jpeg]

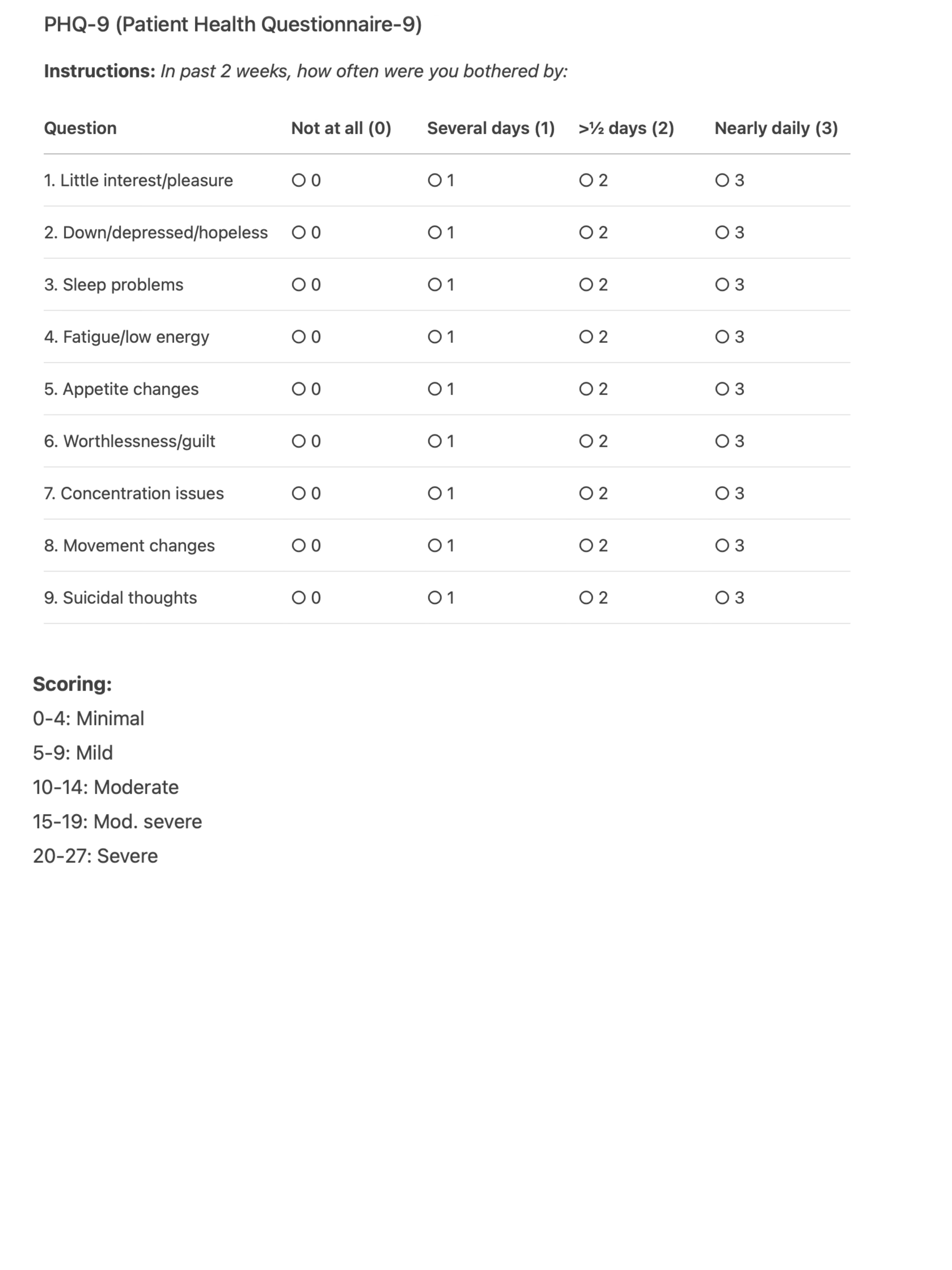

Supplement: Supplementary file 2 [file SupplementaryFile2.jpeg]
